# 

**Published:** 1889-06-22

**Authors:** 


SATURDAY, JUNE 22nd, 1889.

**Congregations and Collections** - 175

**A Lay of ye Ancient Order of St. John of Jerusalem** - 176

**Sighing for his Mother** - 176

**Hospital Sunday Fund.**—Sir Andrew Clark Pleads for the Hospitals - 177

**The Doctor's Pulpit.**—In Nature's School - 179

**Within the Wards.**—Procursive Epilepsy—A Snakebite Cured - 180

**Is Leprosy Infectious** - 181

**Notes and News** - 182

**Everybody's Page** - 184

**Annotations.**—The "Damien" Memorial—The Proposed Pasteur Institute - 185

**The Hospital Workshop.**—No. 3. A Saturday Night at the London Hospital - 186

**Her Heart's Mistake.** By E. D. CUMMING. - 188

**Some Preachers and Subjects for Hospital Sunday** 189

**Amusements and Relaxation** - 190

**Scraps and Gleanings** - 190

EXTRA SUPPLEMENT—THE NURSING MIRROR.

**En Passant.**—Gravesend Hospital—Troubles at Birmingham—In Memoriam—Notes from New York—A Daniel Come to Judgment - xliii
**Whit-Monday in a Children's Hospital**—North Country Customs - xliv
**Nurses on their Travels.**—Farewell to Madeira - xlv
**Everybody's Opinion.**—A Nurses' Club—Nurses' Examinations—Women and their Work—The Food Question xlv
**Appointments.—Vacancies** - xlvi
**Notes and Queries** - xlvi

